# Transition to Online Education during the COVID-19 Pandemic: Impact of Changes in Alcohol Consumption and Experiencing Hangovers on Academic Functioning

**DOI:** 10.3390/jcm10225332

**Published:** 2021-11-16

**Authors:** Agnese Merlo, Pauline A. Hendriksen, Johan Garssen, Elisabeth Y. Bijlsma, Ferdi Engels, Gillian Bruce, Joris C. Verster

**Affiliations:** 1 Division of Pharmacology, Utrecht Institute for Pharmaceutical Sciences, Utrecht University, 3584CG Utrecht, The Netherlands; a.merlo@uu.nl (A.M.); p.a.hendriksen@students.uu.nl (P.A.H.); j.garssen@uu.nl (J.G.); e.y.bijlsma@uu.nl (E.Y.B.); G.M.H.Engels@uu.nl (F.E.); 2 Global Centre of Excellence Immunology, Nutricia Danone Research, 3584CT Utrecht, The Netherlands; 3 Division of Psychology and Social Work, School of Education and Social Sciences, University of the West of Scotland, Paisley PA1 2BE, UK; gillian.bruce@uws.ac.uk; 4 Centre for Human Psychopharmacology, Swinburne University, Melbourne, VIC 3122, Australia

**Keywords:** alcohol, hangover, COVID-19, academic performance, social interactions, students

## Abstract

In the Netherlands, the 2019 coronavirus (COVID-19) pandemic had a significant impact on daily life, with two extensive lockdowns enforced to combat the spread of the SARS-CoV-2 virus. These measures included the closure of bars and restaurants, and the transition from face-to-face to online education. A survey was conducted among Dutch pharmacy students and PhD-candidates to investigate the impact of COVID-19 lockdown on alcohol consumption, hangovers, and academic functioning. The analysis revealed a significant reduction in both quantity and frequency of alcohol consumption during the COVID-19 lockdown periods. This was accompanied with a significant reduction in hangover frequency and lower hangover severity during COVID-19 lockdown periods. The distribution of scores on academic performance showed great variability between respondents: while some participants reported impairment, others reported improved performance during the COVID-19 pandemic, or no change. Women reported that significantly more time investment was associated with maintaining these performance levels. Consistent among participants was the notion of reduced interactions with teachers and other students. Participants who reported more hangovers and most severe hangovers before COVID-19 benefited from the lockdown periods in terms of improved academic performance. Positive correlations were found between study grades/output and both the frequency and severity of hangovers experienced before COVID-19, suggesting that heavier drinkers, in particular, improved academic performance during the lockdown periods. In conclusion, COVID-19 lockdowns were associated with a significant reduction in both alcohol consumption and experiencing hangovers, which was, among heavier drinkers particularly, associated with significantly improved academic functioning.

## 1. Introduction

**Figure 1:**
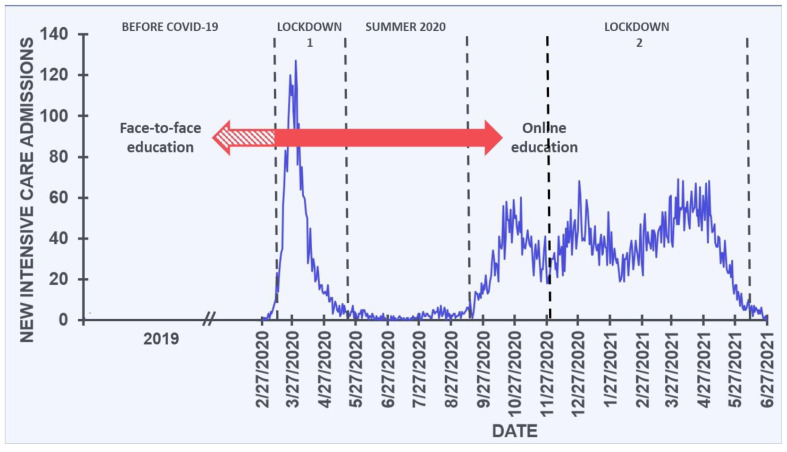
Number of COVID-19 intensive care patients in the Netherlands and related lockdowns. During the lockdown periods, bars and restaurants were closed, and universities switched from face-to-face education to online education. The blue line represents the number of new intensive care admissions. The red arrows represent the periods of predominantly face-to-face education (before mid-March 2020) and predominantly online education (after mid-March 2020). Dashed lines segregate the lockdown and non-lockdown periods. Data from reference [1].

In The Netherlands, the 2019 coronavirus (COVID-19) pandemic had a significant impact on daily life, with two extensive lockdowns enforced to combat the spread of the SARS-CoV-2 virus. In March 2020, the number of people testing positive for SARS-CoV-2 grew exponentially, and the first lockdown period was enforced on 15 March 2020. The first lockdown lasted until May, 2020, and comprised working from home, the closure of universities, bars, restaurants and all non-essential stores. People were instructed to stay at home as much as possible, and for students’ face-to-face education changed to online education. In May 2020, the number of SARS-CoV-2 infections decreased rapidly. After a summer period without lockdown measures, a second lockdown was implemented in November 2020 when the number of positive SARS-CoV-2 cases again rose exponentially. COVID-19 measures were the same as during the first lockdown, but during the second lockdown in addition mandatory wearing face masks at indoor venues and a night curfew were implemented. The lockdown lasted until April 2021 (See Figure 1).

Although in the absence of vaccines, lockdowns were considered an effective way to reduce the spread of the coronavirus, there is a growing body of scientific literature pointing to the negative effects of lockdowns [2,3]. Studies in student samples report that lockdown periods were accompanied by significant decreases in psychological wellbeing, increased stress, loneliness, and depression, and decreased quality of life [2,3]. With regard to students, significant changes in academic and social life accompanied the lockdown periods. During lockdown, both academic activities and social interactions changed from in-person to online events. Research from around the world reported mixed results regarding the transition from in-person to online education. Some studies pointed at the advantages of online teaching such as being more time-effective and reported improved academic performance during the COVID-19 pandemic [4,5,6]. However, other studies reported a negative impact of the COVID-19 pandemic on academic functioning [7,8,9,10,11,12]. These studies for example noted that online teaching activities required greater mental effort and that motivation was compromised, whereas the lockdowns itself were associated with poorer mood and reduced quality of life. Therefore, further research in this area is warranted.

Various coping strategies have been adopted to adapt to the lockdown situation. Amongst others, such as being physically active or adopting a healthy diet, these also included substance abuse. For example, Prowse et al. [13] reported on the increased use of cannabis in both male and female students. In male students the increase in cannabis use was associated with a negative impact on academic performance and increased stress and other mental health complaints. Interestingly, the associations were not significant among female students. Czeisler [14] also reported that the increase in use of cannabis, alcohol and vaping nicotine to cope with COVID-19 related stress was greatest among 18 to 24 years old male individuals.

An increasing number of studies evaluated alcohol consumption during the COVID-19 pandemic, in particular the effects of lockdown. These studies show that alcohol consumption either increased, decreased, or remained the same during the COVID-19 pandemic [15,16,17,18,19,20,21]. Differences may be related to specific sample characteristics (e.g., age), but also differences in the strictness of lockdowns between countries were the research was performed. However, until now, no publications have reported on alcohol hangover in the context of COVID-19. The alcohol hangover is the most frequently reported adverse event of alcohol consumption [22] and is defined as the combination of negative mental and physical symptoms which can be experienced after a single episode of alcohol consumption, starting when blood alcohol concentration (BAC) approaches zero. [23,24]. Hangovers are characterized by a variety of symptoms that may negatively impact academic performance, including fatigue and concentration problems [25,26]. The outcomes of a meta-analysis [27] and critical review [28] showed that cognitive skills and abilities that are relevant for academic performance were significantly impaired during alcohol hangover state, including learning, short- and long-term memory, and sustained attention. These cognitive impairments are translated in impaired performance of daily activities such driving a car [29,30,31] and job performance [32,33,34], and it can be hypothesized that they also will have a negative impact on academic performance.

Previous research conducted before the COVID-19 pandemic that specifically focused on academic performance during alcohol hangover is very limited. In several studies, a negative impact of excessive alcohol consumption on academic functioning has been reported by part of the participants, and this has been related to alcohol hangover. For example, Hallett et al. [35] revealed that in Australia a substantial number of students reported that due to their alcohol consumption they had missed a class (25.6%) or were unable to concentrate in class (25.7%). About half of these students (45%) reported that alcohol consumption negatively impacted their learning or grades and the most frequent reported problem in this context was having hangovers (74.8%). Similarly, of 800 Dutch students that evaluated the negative alcohol-related consequences they experienced over the past year, 28.0% reported missing classes and 21.7% reported the quality of school work suffered due to alcohol consumption, and 74.3% reported having had hangovers [22]. Although it may be tempting to assume that the effects on academic functioning are directly related to alcohol hangover effects, this conclusion cannot be drawn from this data, as the events were not linked to specific dates. In other words, it may be theoretically possible that a hangover was experienced on one day, while missed classes were experienced on a hangover-free day. Until now, only one double-blind crossover study has been conducted to investigate academic performance during alcohol hangover. In this study, Howland et al. [36] examined academic functions of 196 US college students the day following consuming alcohol to achieve a breath alcohol concentration of 0.12% or a placebo drink. On the evening of the drinking session, before beverage consumption participants viewed a 30-min video on a health topic and read a corresponding textbook chapter. Thereafter treatments were administered. Next morning, participants completed a quiz and had to answer questions on the studied topic. In addition, participants completed a graduate record examination, comprising 30 min testing of verbal abilities and 45 min assessing mathematical skills, ability to reason, and solve quantitative problems (e.g., arithmetic and geometric). While participants had consumed a considerable amount of alcohol the day before, no significant differences between the hangover and control day were found on the quiz and graduate record examination.

### 1.1. Educational Changes during the COVID-19 Pandemic

Teaching at the department of pharmaceutical sciences of Utrecht University is mainly centered around small-scale student-centered and context-rich teaching formats with a strong focus on collaborative learning, such as problem-based learning (case-based learning) and project-and inquiry-based learning. These types of classes are supplemented with lectures and practical course work. Before the start of the pandemic, most teaching was done on campus, although some courses had already implemented some form of computer-supported learning for e.g., self-study and peer-feedback assignments. In line with other universities around the world, soon after the start of the COVID-19 pandemic, an overnight change had to be made from in-person teaching activities to online teaching.

On the 11th of May 2020, the lockdown ended and universities partially reopened. However, social distancing measures were still enforced and as a result most teaching activities remained online. Throughout the summer of 2020 there were no major COVID-19 outbreaks in The Netherlands. Academic activities were minimal during the summer and most students enjoyed the holiday season. After summer (September 2020) limited on campus education was re-instated, where universities aimed at welcoming students on campus for practical course work, research internships and once a week for regular course work. From September onward the number of positive COVID-19 cases rose again, and a second lockdown was installed in November (2020). This lockdown lasted until April 2021. During the second lockdown regular classes switched to online education again, whereas practical course work and research internships continued on campus.

### 1.2. Research Aim and Hypotheses

Given the current lack of knowledge, the aim of the current study was to investigate the relationship of alcohol consumption and having hangovers with academic performance during COVID-19 lockdowns among Dutch pharmacy students and PhD-candidates. In addition, the impact of the first COVID-19 lockdown on social interactions with teachers and other students was also examined. It is hypothesized that changes in alcohol consumption (quantity and frequency) and the frequency and severity of hangovers during lockdowns will also be associated with impairments in academic performance.

## 2. Materials and Methods

An online survey was conducted in the first week of June 2021 among students and PhD-candidates of the department of pharmaceutical sciences of Utrecht University, The Netherlands. The study was reviewed and approved by the Science-Geo Ethics Review Board of Utrecht University (protocol code: S-21525, date of approval: 19 May 2021), and all participants gave electronic informed consent. The survey was designed via SurveyMonkey and took about ten minutes to complete. As an incentive participants could enter a prize draw to wine one of two Euro 100 vouchers. A thorough description of the study methodology and the forthcoming dataset have been published elsewhere [37].

Participants of the study were students, PhD-candidates, and postdocs from the department of Pharmaceutical Sciences of Utrecht University, the Netherlands. The department of Pharmaceutical Sciences is responsible for one of the three Pharmacy programs in the Netherlands. Pharmacy students first follow a three-year Bachelor program, most-often followed by a Master program comprising three more years of education, resulting in a PharmD degree. Within the Bachelor of Pharmaceutical Sciences, the department hosts a research-oriented international program, the College of Pharmaceutical Sciences (CPS). In addition, the Utrecht Institute of Pharmaceutical Sciences (UIPS), the research institute of the department, offers a PhD program in Drug Innovation and is responsible for the further development of postdoctoral researchers. In the academic year 2020–2021, 662 students followed the bachelor pharmaceutical sciences and 142 students the College of Pharmaceutical Sciences. A total of 458 students were registered for the master, and 190 PhD-candidates and 30 postdocs were affiliated with UIPS. Since the department comprises a considerable number of international students, participants could choose to complete the survey in English or Dutch language.

### 2.1. Survey Content

Retrospective assessments of alcohol consumption were made for (1) the year 2019 (the period before COVID-19), (2) the first lockdown period, (3) summer 2020 (no lockdown), and (4) the second lockdown (November 2020 -March 2021). Changes in academic functioning were assessed for the COVID-19 pandemic as a whole, in comparison to the situation before the start of the COVID-19 pandemic.

#### 2.1.1. Demographics

Questions assessing demographics includes age, sex, weight, and height. Participants indicated whether they were a student, PhD-candidate or postdoc, and whether they lived alone, or together with family or others (e.g., students or friends) during the COVID-19 pandemic.

#### 2.1.2. Alcohol Consumption

Questions about alcohol consumption included the number of alcoholic drinks participants consumed on average per week, and the number of days per week they consumed alcohol. Guidance was provided on serving sizes and how to convert these alcoholic drinks into units. Participants further reported how many hangovers they experienced and the average severity of their hangovers. Hangover severity was assessed on an 11-point scale ranging from 0 (absent) to 10 (extreme) [38]. The questions were completed for (1) the year 2019 (the period before the lockdown), (2) the first lockdown period, (3) summer 2020 (no lockdown), and (4) the second lockdown (November 2020 -April 2021).

#### 2.1.3. Academic Functioning

The academic functioning scale was specifically designed for this study. Participants rated, compared to before COVID-19, their study/work performance during the COVID-19 pandemic on scales ranging from -5 (extremely worse) to +5 (extremely better), around a midpoint of 0 (unchanged). Six items relate to academic performance and include ‘Quality’ (“overall performance quality”), ‘Time’ (“amount of time invested in study/PhD-/postdoc project”), ‘Grades/Output’ (“study grades/output”), ‘Knowledge’ (“academic achievement/amount of knowledge gained”), ‘Reading’ (“reading articles/text books”), and ‘Writing’ (“writing assignments/ articles”)“. Two items relate to academic interactions and include ‘Contact with teachers’ (“contact with teachers or supervisors”) and ‘Interactions with students’ (“interactions with other students/PhD-candidates/postdocs”). Finally, two other items relate to satisfaction with academic life and include ‘Balance study-private life’ (“balance between work/study and private life”) and ‘Role-satisfaction’ (“the extent you enjoy being a student/PhD-candidate/postdoc”).

#### 2.1.4. Statistical Analysis

Data were analyzed with SPSS (IBM Corp. Released 2013. IBM SPSS Statistics for Windows, Version 27.0. IBM Corp., Armonk, NY, USA). Mean and standard deviation (SD) were computed for all variables and distributions were checked for normality with the Kolmogorov–Smirnov test and by visual inspection. Data were not normally distributed, and therefore nonparametric tests were conducted for the statistical analysis. For the purpose of the current study, only data from participants that consume alcohol was considered for the statistical analysis.

Within-subject comparisons to compare assessments that are made for four timepoints (2019, first lockdown, summer, second lockdown) were conducted with the Related-Samples Friedman’s Two Way Analysis of Variance by Ranks test. A Bonferroni’s correct was applied to adjust the *p*-value for multiple comparisons. Differences between the assessments were considered significant if the adjusted *p* value was <0.05. Between-group comparisons to evaluate sex differences were conducted with the Independent Samples Mann-Whitney U test. Differences between the groups were considered significant if *p* < 0.05, and a Bonferroni correction was applied for multiple related comparisons. Finally, Spearman’s correlations were computed between the Δ scores for alcohol consumption outcomes (the average score during the two COVID-19 lockdowns–before COVID-19) and academic functioning outcomes. Correlations were considered significant if *p* < 0.05, and a Bonferroni correction was applied to account for multiple comparisons.

## 3. Results

Of the *n* = 1452 students that were invited, *n* = 341 started the survey (response rate: 23.5%), and *n* = 250 completed the survey. Of these, *n* = 156 participants reported consuming alcohol and were included in the statistical analyses. *n* = 94 served as a control group (see Section 3.1). Demographics of the participants that consumed alcohol are summarized in Table 1.

Female students were slightly older than male students (*p* = 0.037). No significant sex differences were found for student status or living situation. Table 2 summarizes their alcohol consumption outcomes assessed for before and during the COVID-19 pandemic.

**Figure 2:**
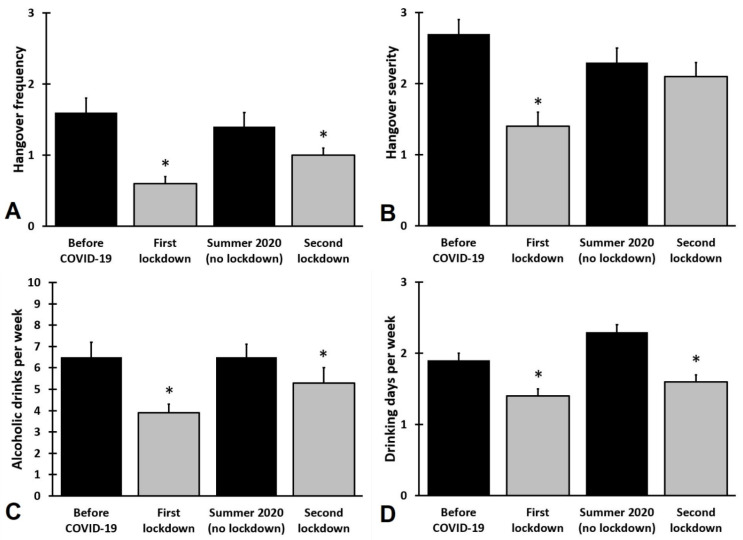
The effect of the COVID-19 pandemic on alcohol consumption and hangovers. Mean and standard errors are shown for hangover frequency (**A**), hangover severity (**B**), alcoholic drinks consumed per week (**C**), and the number of drinking days per week (**D**). Significant differences (adjusted *p*-values < 0.05, applying a Bonferroni’s correction for multiple comparisons) from ‘before COVID-19) are indicated by *.

The distribution of changes in weekly alcohol consumption and monthly hangover frequency shows that there is also a subgroup of students that increased alcohol intake during the lockdown periods. In the current sample, 56.3% reduced weekly alcohol intake, 17.9% consumed the same amount of alcohol, and 25.8% increased weekly alcohol intake during the COVID-19 lockdowns. In line, 47.4% reported a reduced hangover frequency, 39.5% reported the same hangover frequency, and 13.5% reported increased hangover frequency during the COVID-19 lockdowns. Overall, a significant reduction was seen in quantity and frequency of weekly alcohol consumption and corresponding monthly hangovers during the two lockdown periods (See Figure 2). The ratings for summer 2020 (no lockdown) did not significantly differ from before COVID-19.

For all time periods, women consumed significantly less alcohol per week than men (*p* < 0.001 for before COVID-19 and the first lockdown, *p* = 0.01 for summer 2020 and *p* < 0.005 for the second lockdown). For women, the number of drinking days per week was also lower than in men for each time period, but after Bonferroni’s correction this difference reached statistical significance only for the first lockdown period (*p* = 0.012). In line, the average hangover severity reported by women was significantly lower than that reported by men for both the first lockdown period (*p* < 0.001) and second lockdown period (*p* = 0.009). Hangover frequency was also lower in women compared to men, with a significant difference found for the first lockdown period (*p* = 0.002).

**Figure 3:**
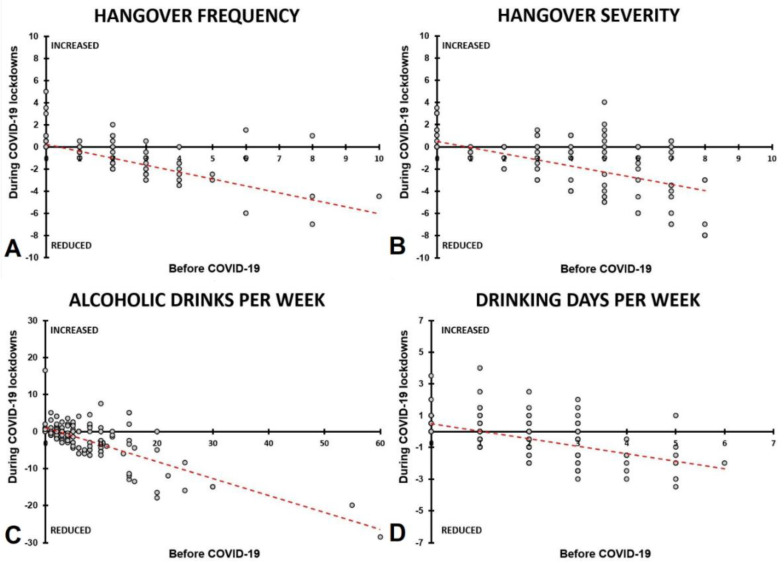
The effect of COVID-19 lockdowns on alcohol consumption and hangovers. Spearman’s correlations are shown between assessments made for ‘before COVID-19′ and during COVID-19 lockdowns. The change from before COVID-19 (i.e., the average score of the two lockdown periods-the ‘before COVID-19′ assessment) was correlated with the ‘before COVID-19′ assessment. Correlations are shown for hangover frequency (**A**), hangover severity (**B**), alcoholic drinks consumed per week (**C**), and the number of drinking days per week (**D**). All correlations were significant (*p* < 0.001).

The reduction in hangover severity and frequency during COVID-19 lockdowns was largest among those who reported the highest levels of hangover frequency and severity before COVID-19. The correlations between assessments made for ‘before COVID-19′ and ‘during COVID-19 lockdowns’ were highly significant for hangover frequency (overall: r = −0.712, *p* < 0.001; men: r = −0.717, *p* < 0.001; women: r = −0.713, *p* < 0.001) and hangover severity (overall: r = −0.559, *p* < 0.001; women: r = −0.688, *p* < 0.001) (See Figure 3A,B). For men, the correlation hangover severity before COVID-19 and the reduction in hangover severity during COVID-19 did not reach significance (r = −0.300, *p* = 0.068). Similarly, reduction in alcoholic drinks consumed and number of drinking days per week during COVID-19 lockdowns was largest among those who reported the highest levels of alcohol consumption before COVID-19. The correlations between assessments made for ‘before COVID-19′ and ‘during COVID-19 lockdowns’ were highly significant for both the amount of alcohol consumed (overall: r = −0.490, *p* < 0.001; men: r = −0.484, *p* = 0.002; women: r = −0.526, *p* < 0.001) and the number of drinking days per week (overall: r = −0.471, *p* < 0.001; men: r = −0.414, *p* = 0.010; women: r =−0.498, *p* < 0.001) (See Figure 3C,D). In other words, the effect of lockdowns on alcohol consumption outcomes was most pronounced in participants that were heavy drinkers before the COVID-19 pandemic compared to modest drinkers.

**Figure 4:**
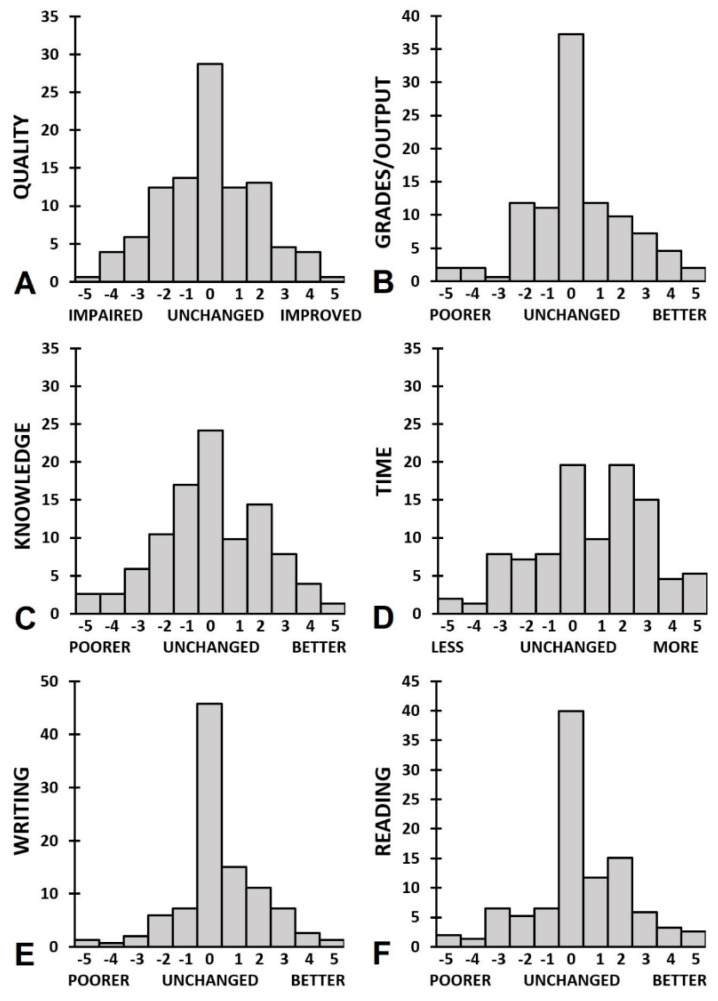
The impact of the COVID-19 pandemic on academic performance. The distributions of percentages endorsement are shown for (**A**) overall performance quality, (**B**) study grades/output, (**C**) knowledge, (**D**) time, (**E**) writing, and (**F**) reading.

**Figure 5:**
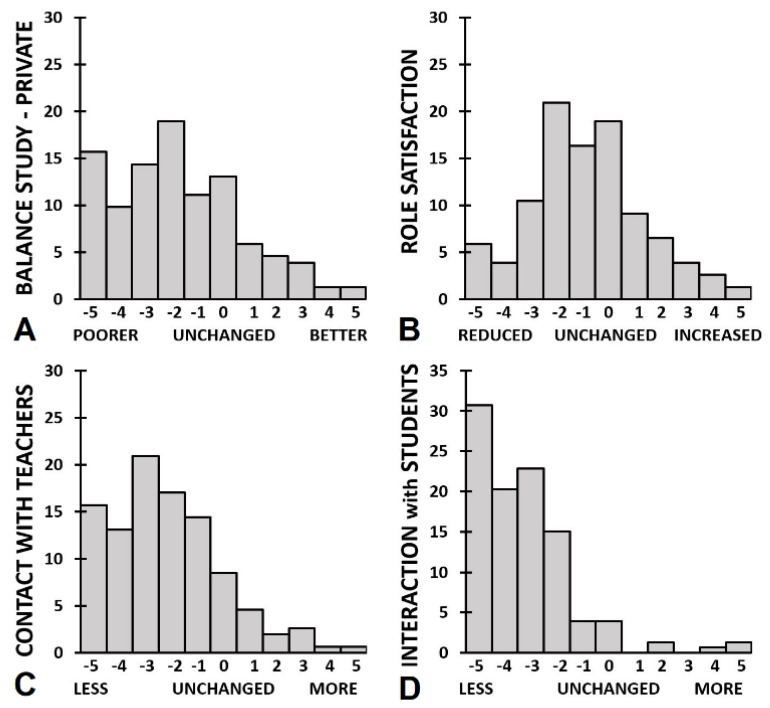
The impact of the COVID-19 pandemic on academic interactions. The distribution of percentages endorsement are shown for, and (**A**) balance study-private life, (**B**) role satisfaction, (**C**) contact with teachers, and (**D**) interactions with students.

Academic functioning outcomes for men and women are summarized in Table 3 and Figure 4 and Figure 5. Figure 4 shows that differential effects on academic performance were reported: both improvement and impairment was reported by participants. Figure 5 shows that the majority of participants reported a reduction in contact with teachers and interactions with other students. Statistical analysis revealed that men reported a significant reduction in contact with teachers or supervisors (*p* < 0.001) and interactions with other (PhD-) students (*p* < 0.001). The distorted balance between work/study and private life (*p* = 0.009) did not reach statistical significance after a Bonferroni’s correction was applied. In women the effects were of the same magnitude and in the same direction as observed in men. However, due to the larger sample size, in women more items reached statistical significance (e.g., time and writing). None of the direct comparisons between men and women reached statistical significance.

Table 4 summarizes the correlations between alcohol consumption and academic functioning. For the sample as a whole, a significant correlation (*p* < 0.007 after Bonferroni’s correction) was found between study grades/output and hangover frequency (*p* = 0.005).

In women, a significant correlation was found between grades/output and hangover frequency (*p* = 0.005). The correlations between time and hangover frequency (*p* = 0.008) approached significance. In men, none of the correlations reached statistical significance.

**Figure 6:**
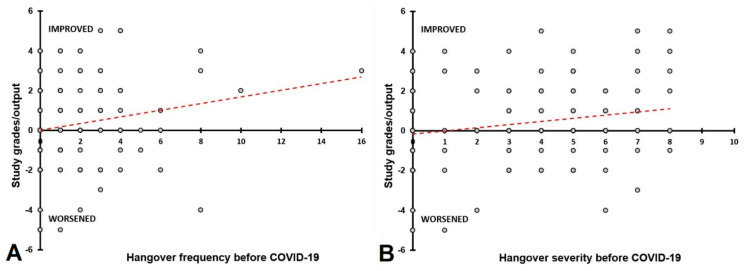
Relationship between having hangovers before COVID-19 and study grades/output during the COVID-19 pandemic. Spearman’s correlations are shown between hangover assessments made for ‘before COVID-19′ and study grades/output during the COVID-19 pandemic (the difference score relative to ‘before COVID-19′). Correlations are shown for hangover frequency (**A**) and hangover severity (**B**).

Again, participants who reported most hangovers and most severe hangovers before COVID-19 benefit most from the lockdown periods in terms of academic performance. Positive correlations were found between study grades/output and the both the frequency (r = 0.206, *p* = 0.011) and severity (r = 0.179, *p* = 0.027) of hangovers experienced before COVID-19, suggesting that in particular the heavier drinkers improved academic performance during the lockdown periods (see Figure 6).

### 3.1. Comparison with Participants That Not Consume Alcohol

A total of *n* = 94 participants did not consume alcohol and were omitted from the previous analyses. Their demographics are summarized in Table 5. No significant differences were found between the groups, except for living situation (*p* < 0.001) revealing that those who consume no alcohol significantly more frequently live together with family, whereas those that consume alcohol most frequently live to together with friends.

Academic functioning outcomes of both groups are summarized in Table 6. No significant differences were found between the groups.

## 4. Discussion

The analysis revealed a significant reduction in both quantity and frequency of alcohol consumption during the COVID-19 lockdown periods. This was accompanied by a significant reduction in hangover frequency and lower hangover severity. These effects were especially robust among female participants. Due to the relatively small sample size the differences did not always reach statistical significance among male participants, although they did point in the same direction.

Overall, academic performance was not affected during the COVID-19 pandemic. However, the distribution of scores on academic performance show great variability between respondents: while part of participants reported impairment, others reported improved performance during the COVID-19 pandemic, or no change (see Figure 4). Women reported that significantly more time investment was associated with maintaining these performance levels. This effect was not seen among male participants. In future studies, it is important to further investigate demographics and other characteristics of students that either improve or impair their performance, and those that seem unaffected by lockdowns. Profiling students that are at risk for poorer academic performance will help direct prevention and support to those students that need it in case of future lockdowns.

Both male and female participants reported a significant reduction in contacts with teachers and interactions with other students. Further, female participants reported a significant reduction in the balance between study and private life, and an overall reduction of role satisfaction. In contrast to differential distribution of lockdown effects on academic performance, there were only few participants that reported that social interactions improved during the COVID-19 lockdowns. Instead, the vast majority reported a reduction in these interactions (See Figure 5).

The largest reduction in alcohol consumption and experiencing hangovers was observed among participants who reported the greatest alcohol intake before COVID-19 (see Figure 3). The data further show that participants who reported the most frequent and severe hangovers before the COVID-19 pandemic benefited most from the lockdowns in terms of improving their study grades/output (see Figure 6).

Our findings are in line with other studies in that subsamples exist that either increased or decreased alcohol consumption during COVID-19 lockdowns [15,16,17,18,19,20,21]. Overall, a significant reduction was found in weekly alcohol consumption and hangover frequency. This is in line with recent research from the Netherlands, also showing a reduction of alcohol intake among young adults [39,40]. With regard to academic performance a similar diverse distribution was seen, including participants with improved or worsened academic functioning. Overall, academic performance improved, and social interactions were significantly reduced during the COVID-19 lockdowns. When comparing different studies, it is important to realize that different outcomes may be related to specific sample characteristics (e.g., age), but also differences in the strictness of lockdowns between countries where the research was performed may play a role. Of note in this context, in the current study there were differences in measures between the first and second lockdown, such as waring facemasks and the introduction of a night curfew in the second lockdown. These measures may have affected both alcohol consumption and academic performance and might in part explain differences observed between the first and second lockdown period. However, we think that, if any, the impact of the night curfew had a relatively small impact on alcohol consumption as the venues where alcohol could be consumed (bars, restaurants) were closed during both the first and second lockdown period. In addition, restrictions were enforced regarding the number of guests one could invite at home, preventing nightlife entertainment to be replaced by private parties.

The outcomes should be interpreted by taking into account the study’s limitations and strengths. Firstly, the data were collected retrospectively. Therefore, recall bias may have influenced the study outcomes. Secondly, the questionnaire assessing academic functioning was developed in-house. We choose to use this questionnaire as it was short and to our opinion captured the most important aspects of academic performance and social interactions. To avoid having a lengthy survey, we decided not to use traditional more elaborate questionnaires. Third, the survey was conducted among Dutch pharmacy students. It is therefore unclear to what extent the findings can be generalized to other students in the Netherlands, and internationally. It was decided not to recruit participants from other departments, as the educational methodologies between the departments differ, and this could interfere with the interpretation of study outcomes. Fourth, much more women took part in the study than men. The obtained male/female ratio is however in correspondence with the male/female ratio of the department of pharmaceutical sciences as a whole, as well as most other departments at Utrecht University. It will also be interesting to compare our findings with prospective studies. This will also allow to determine the possible impact of retrospective reporting on the study outcomes. Finally, the current data suggest that, in addition to alcohol consumption, it may be even more important to consider the impact of frequency and severity of alcohol hangover on academic functioning. Therefore, we advocate for more research on the impact of alcohol consumption and hangovers on academic functioning.

## 5. Conclusions

Although a proportion of students have reported increased alcohol consumption and poorer academic functioning, overall, the COVID-19 lockdowns were associated with a significant reduction in both alcohol consumption and experiencing hangovers, which was, in particular among heavier drinkers, associated with significantly improved academic functioning.

## Figures and Tables

**Table 1:** Demographics.

Variable	Overall	Men	Women
*n*	156	38	118
Age (years)	23.9 (4.4)	22.3 (4.1)	22.7 (4.2) *
Living situation			
Alone	18 (11.5 %)	6 (15.8%)	12 (10.2%)
Together with others (students, friends)	83 (53.2 %)	8 (47.4%)	65 (55.1%)
Together with family	55 (35.3 %)	14 (36.8%)	41 (34.7%)
Educational level			
—Bachelor pharmacy or CPS	83 (53.2 %)	16 (42.1%)	67 (56.8%)
—Master pharmacy	53 (34.0 %)	13 (34.2%)	40 (33.9%)
—PhD-candidate	20 (12.8 %)	9 (23.7%)	11 (9.3%)

Results for age are presented as mean and standard deviation (between brackets); other variables as number and percentage (between brackets). Significant sex differences (*p* < 0.05) are indicated by *. Abbreviation: CPS = College of Pharmaceutical Sciences.

**Table 2:** Alcohol consumption.

Variable	Before COVID-19	First Lockdown	Summer 2020 (No Lockdown)	Second Lockdown
Alcoholic drinks per week				
Overall	6.5 (8.5)	3.9 (5.2) ^a^	6.5 (7.0) ^b^	5.3 (8.2) ^a,c^
Men	11.2 (11.3)	7.3 (6.9)	10.1 (10.0)	8.5 (10.6)
Women	5.0 (6.7) *	2.8 (3.9) *^,a^	5.3 (5.1) *	4.2 (7.0) *^,b,c^
Drinking days per week				
Overall	1.9 (1.3)	1.4 (1.5) ^a^	2.3 (1.4) ^b^	1.6 (1.4) ^a,c^
Men	2.4 (1.4)	1.8 (1.5)	2.5 (1.4)	2.0 (1.5) ^a,c^
Women	1.8 (1.2)	1.3 (1.5) *^,a^	2.3 (1.4) ^b^	1.5 (1.4) ^c^
Hangover severity				
Overall	2.7 (2.7)	1.4 (2.3) ^a^	2.3 (2.7) ^b^	2.1 (2.7)
Men	3.4 (2.7)	2.7 (2.7)	3.3 (2.9)	3.1 (2.8)
Women	2.5 (2.7)	0.9 (2.0) *^,a^	2.0 (2.6) ^b^	1.7 (2.5) *
Hangover frequency				
Overall	1.6 (2.2)	0.6 (1.4) ^a^	1.4 (2.2) ^b^	1.0 (1.8) ^a^
Men	2.1 (2.5)	0.9 (1.2) ^a^	1.8 (2.2)	1.4 (2.2)
Women	1.4 (2.1)	0.5 (1.5) *^,a^	1.2 (2.2) ^b^	0.9 (1.6) ^a^

Mean (SD) are shown. Significant sex differences (*p* < 0.0125) are indicated by *. Significant differences (adjusted *p*-values <0.05, applying a Bonferroni correction for multiple comparisons) between time periods are indicated as follows: a = significantly different from ‘before COVID-19′, b = significant difference from the ‘first lockdown’, c = significant difference from ‘summer 2020′.

**Table 3:** Academic functioning during the COVID-19 pandemic according to sex.

Academic Functioning	Men	Women
Quality	−0.1 (1.9)	0.0 (2.0)
Time	0.1 (2.5)	1.0 (2.3) *
Grades/Output	0.3 (1.9)	0.4 (2.1)
Knowledge	0.2 (2.3)	0.2 (2.3)
Reading	0.3 (2.0)	0.4 (2.0)
Writing	0.2 (2.0)	0.5 (1.7) *
Contact with teachers	−2.0 (2.2) *	−2.0 (2.2) *
Interactions with students	−2.9 (2.2) *	−3.0 (2.0) *
Balance study-private life	−1.0 (2.8)	−1.7 (2.4) *
Role satisfaction	−0.6 (2.3)	−0.6 (2.4) *

Mean (SD) are shown. Significant changes relative to ‘before COVID-19′ (*p* < 0.005, after Bonferroni’ correction for multiple comparisons) are indicated by *. No significant differences between men and women were found.

**Table 4:** Relationship between alcohol consumption and academic functioning.

Variable	Alcoholic Drinks per Week	Drinking Days per Week	Hangover Severity	Hangover Frequency
Quality				
Overall	−0.067	−0.014	−0.043	−0.084
Men	−0.203	−0.061	0.066	0.028
Women	0.002	−0.001	−0.099	−0.129
Time				
Overall	−0.025	−0.007	−0.119	−0.167
Men	−0.077	0.016	0.066	0.028
Women	−0.020	−0.024	−0.179	−0.248
Grades/Output				
Overall	−0.157	−0.073	−0.086	−0.225 *
Men	−0.253	−0.074	0.095	−0.139
Women	−0.123	−0.077	−0.144	−0.261 *
Knowledge				
Overall	−0.076	0.023	−0.009	−0.066
Men	−0.138	−0.011	0.134	0.009
Women	−0.056	0.035	−0.057	−0.086
Reading				
Overall	0.065	0.150	0.026	−0.027
Men	0.124	0.256	0.005	0.048
Women	0.032	0.114	0.027	−0.052
Writing				
Overall	−0.012	0.028	−0.066	−0.076
Men	−0.086	0.014	0.038	−0.175
Women	−0.011	0.018	−0.088	−0.053
Contact with teachers				
Overall	−0.004	0.009	0.073	0.037
Men	−0.024	−0.053	0.025	0.064
Women	0.004	0.032	0.077	0.051
Interactions with students				
Overall	−0.171	0.189	0.030	0.098
Men	0.262	0.200	0.028	0.215
Women	0.143	0.196	0.012	0.075
Balance study-private life				
Overall	0.052	0.053	0.114	0.007
Men	0.253	0.273	0.159	0.105
Women	0.004	0.007	0.101	0.022
Role satisfaction				
Overall	0.036	0.079	0.091	0.010
Men	−0.072	0.098	0.159	0.050
Women	0.084	0.070	0.058	−0.001

Spearman’s correlations between difference scores (average score of the two COVID-19 lockdowns– before COVID-19) are shown. Significant correlations (*p* < 0.007, after Bonferroni’ correction) are indicated by *.

**Table 5:** Demographics.

Variable	Alcohol	No Alcohol
*n*	156	94
Age (years)	23.9 (4.4)	22.4 (3.3)
Living situation		
Alone	18 (11.5 %)	11 (11.7%)
Together with others (students, friends)	83 (53.2 %)	18 (19.1%) *
Together with family	55 (35.3 %)	65 (69.1%) *
Educational level		
—Bachelor pharmacy or CPS	83 (53.2 %)	60 (63.8%)
—Master pharmacy	53 (34.0 %)	23 (24.5%)
—PhD-candidate	20 (12.8 %)	11 (11.7%)

Results for age are presented as mean and standard deviation (between brackets); other variables as number and percentage (between brackets). Significant differences between the alcohol and no alcohol group (*p* < 0.05) are indicated by *.

**Table 6:** Academic functioning of participants that did or did not consume alcohol during the COVID-19 pandemic.

Academic Functioning	Alcohol	No alcohol
Quality	−0.4 (2.0)	0.10 (2.1)
Time	0.78 (2.3) *	0.69 (2.5)
Grades/Output	0.27 (1.9)	0.61 (2.2)
Knowledge	0.05 (2.1)	0.46 (2.6)
Reading	0.34 (2.0)	0.43 (2.1)
Writing	0.42 (1.7) *	0.43 (2.0)
Contact with teachers	−2.20 (2.1) *	−1.82 (2.2) *
Interactions with students	−3.25 (1.9) *	−2.57 (2.3) *
Balance study-private life	−1.71 (2.4) *	−1.28 (2.7) *
Role satisfaction	−0.84 (2.2) *	−0.17 (2.6)

Mean (SD) are shown. Significant changes relative to ‘before COVID-19′ (*p* < 0.005, after Bonferroni’ correction for multiple comparisons) are indicated by *. No significant differences between the alcohol and no alcohol group (*p* < 0.005, after Bonferroni’ correction for multiple comparisons) were found.

## Data Availability

The data is published open access in the journal MDPI Data and available online as supplement to reference [37].
